# 2D Nano-Mica Sheets Assembled Membranes for High-Efficiency Oil/Water Separation

**DOI:** 10.3390/nano12172895

**Published:** 2022-08-23

**Authors:** Yan Bao, Bin Wang, Conghui Du, Qiuhui Shi, Wenlong Xu, Zhining Wang

**Affiliations:** 1 Shandong Key Laboratory of Environmental Processes and Health, School of Environmental Science and Engineering, Shandong University, Qingdao 266237, China; 2 Qingdao Institute of Bioenergy and Bioprocess Technology, Chinese Academy of Sciences, Qingdao 266101, China; 3 Informatization Office, Shandong University, Ji’nan 250100, China

**Keywords:** oil/water separation, emulsions, nano-mica sheets, 2D nanomaterial composite membranes, super-oleophobicity

## Abstract

Oil-polluted water has become one of the most important environmental concerns nowadays due to the increasing industrial oily wastewater and frequent oil spill accidents. Herein, a novel two-dimensional (2D) nano-mica sheets assembled composite membrane with underwater super-oleophobic properties was developed for effective oil/water separation. A 2D nano-mica sheet was synthesized by a facile solvent-assisted ultrasonic exfoliation and then the obtained 2D nano-mica sheets were co-deposited with dopamine on polyvinylidene fluoride substrate to prepare nano-mica composite membranes (NCM). The NCM is hydrophilic in air and super-oleophobic underwater (the water contact angle in the air is 37.6°, and the oil contact angle in water is 151.4°). Furthermore, the prepared NCM provided outstanding stability in different acid–base environments (pH = 1–11). Noteworthily, the oil removal rate is higher than 99.5% as the sodium dodecyl sulfate SDS-stabilized oil (soya-bean oil, mineral oil and pump oil) -in-water emulsions. Meanwhile, the NCM showed excellent reusability, as the oil removal efficiency kept at 99.0% after ten soya-bean oil-in-water or mineral oil-in-water emulsion filtration cycles. The present work paved a new way for developing a low-cost and environmentally friendly strategy for oily wastewater treatment and developed a high-increment utilization application field for natural minerals.

## 1. Introduction

Oil/water separation is among the most important challenges with the ever-growing energy consumption and rapid industrialization, as well as the frequent oil spill accidents [1,2,3]. Considerable amounts of oily wastewater are produced daily from petrochemical, textile, metallurgy, steel, and other industries, causing serious threats to the environment and ecological systems [4,5]. The oil floating on the ocean surface will quickly spread into an impermeable oil film, which hinders the reoxygenation of water, leads to hypoxia of ocean water, affects the growth of marine plankton, and even destroys the ecological balance of the ocean [5].

Oily wastewater can be divided into four types according to the physical state: free oil, dispersed oil, dissolved oil, and emulsified oil [6]. The separation of emulsified oil/water mixtures remains a major challenge because the oil is stably dispersed in the water through surfactant to form the oil-in-water emulsion [7]. At present, various strategies, such as gravity settling, air flotation, centrifugal separation, three-dimensional material absorption or adsorption, and membrane separation, have been enrolled in oil/water separation [8,9,10,11,12,13,14]. Among the different methods, membrane separation has been attracting immense attention for its high separation efficiency, cost-effectiveness, low energy consumption, versatility, and low environmental pollution [15,16].

Membrane separation has been successfully applied in desalination and wastewater treatment, according to its outstanding technical and economic value [17]. Polyvinylidene fluoride (PVDF) has excellent heat resistance, good mechanical strength and porosity, and is a promising polymer material as a water treatment membrane. However, the inherent hydrophobicity of PVDF membranes can lead to serious organic pollution, reduce the filtration efficiency and shorten the service life of the membrane, which limits its application in practical water treatment [18]. The essence of oil/water separation is an interfacial issue. It requires developing materials with the super-wetting surface to achieve highly efficient oil/water separation [19,20]. Oil/water separation membranes based on special wettability can be divided into two categories: super-hydrophobic/super-oleophilic and super-hydrophilic/underwater super-oleophobic [9,21]. For the super-hydrophobic/super-oleophilic membrane surface, oil droplets quickly wet the surface and enable oil to continuously permeate the structure, while blocking water for it cannot wet the film surface [22]. However, the membranes are severely affected by membrane fouling due to its potential oleophilic nature, oil droplets, and other impurities that are easily and irreversibly adsorbed on the surface, leading to serious contamination and flux decline [23]. In addition, the oil droplets tend to coalesce together and rapidly absorb onto the membrane surface during cleaning, therefore resulting in a shortened lifespan and increased energy consumption [24]. These disadvantages limit the suitability of super-hydrophobic membranes for the effective separation of oil/water mixtures. On the contrary, a super-hydrophilic and underwater super-oleophobic membrane provides a “water-removing” strategy. The membrane is super-oleophobic in air or underwater, and shows very low adhesion to oil, which effectively prevents the adhesion of oil droplets [25]. When the oily wastewater contacts the surface of the membrane, water droplets can wet the surface quickly and enable water to penetrate the membrane continuously while the oil is trapped on the surface to achieve the oil/water separation effect. Due to the potential oil-repellent property, the oil cannot pollute the membrane surface. This membrane has excellent antifouling properties, low energy consumption, and high separation efficiency, thus super-hydrophilic membrane has been accepted as a promising oil/water separation technique.

Novel nanomaterials offer great opportunities to develop high performance oil/water separation membranes. One-dimensional (nanowires, rods, and tubes) and two-dimensional (layers and sheets) nanomaterials have been widely used to fabricate membranes, due to their excellent properties [26]. The limitation of nanomaterials-based membranes is the complex preparation procedure and the high cost induced by the raw materials. Hence, developing alternative materials with high performance and low cost is critically important [20]. Among various materials, two-dimensional (2D) material has sparked increasing attention as alternative material due to its excellent chemical stability, high mechanical strength, and a large specific surface area [27,28]. Recently, natural ground mica has been successfully stripped into single-layer or few-layer nano-sheets [29]. It has been widely used in the electronic field for its good light transmission, ultra-violet shielding, atomic level flatness, electrical insulation, temperature stability, and chemical durability [30]. However, mica has not been applied in the field of oil/water separation so far. As such, nano-mica sheets based on superior properties (such as good hydrophilicity, good stability, certain rigidity, and ease to obtain) hold the promise of being widely adopted in membrane separation.

Herein, an underwater super hydrophilic nano-mica composite membrane (NCM) was prepared on polyvinylidene fluoride (PVDF) substrate by vacuum-assisted filtration. The addition of 2D nano-mica sheets can modify the PVDF membrane with hydrophobic in air and lipophilic in water into the NCM with hydrophilic in air and super-oleophobic in water. The NCM has excellent oil/water separation performance for SDS-stabilized oil-in-water emulsion. Moreover, it exhibits outstanding stability in water. This work provides a simple and prospective avenue for high added value applications of natural minerals in oil/water separation.

## 2. Materials and Methods

### 2.1. Materials

Nature mica powder (~55 μm) was obtained from Chuzhou Gerui Co., Ltd., Chuzhou, China. The Polyvinylidene fluoride (PVDF) microfiltration support membranes (0.22 μm pore size, 50 mm diameter) were purchased from Yibo Filter Equipment Factory, Haining, China. Dichloromethane, oil red, absolute ethanol, nitric acid (HNO_3_), hydrochloric acids (HCl), sodium hydroxide (NaOH), Sodium dodecyl sulfonate (SDS), cetyltrimethyl ammonium bromide (CTAB), sodium chloride (NaCl), mineral oil were purchased from Sinopharm Chemical Reagent Co., Ltd., Shanghai, China. Dopamine (DA, >99.8%) was purchased from Aladdin (Shanghai) Co., Ltd., Shanghai, China. Tris buffer solution was purchased from Biosharp Co., Ltd., Guangzhou, China. Soya-bean oil was purchased from Yihai Kerry Arawana Holdings Co., Ltd., Qingdao, China. Pump oil was purchased from Huayuan Petrochemical Co., Ltd., Fujian (China). Deionized water was processed from the laboratory pure water device. Without specific notes, all chemicals and materials were used as received.

### 2.2. Preparation of 2D Nano-Mica Sheets

**Figure 1:**
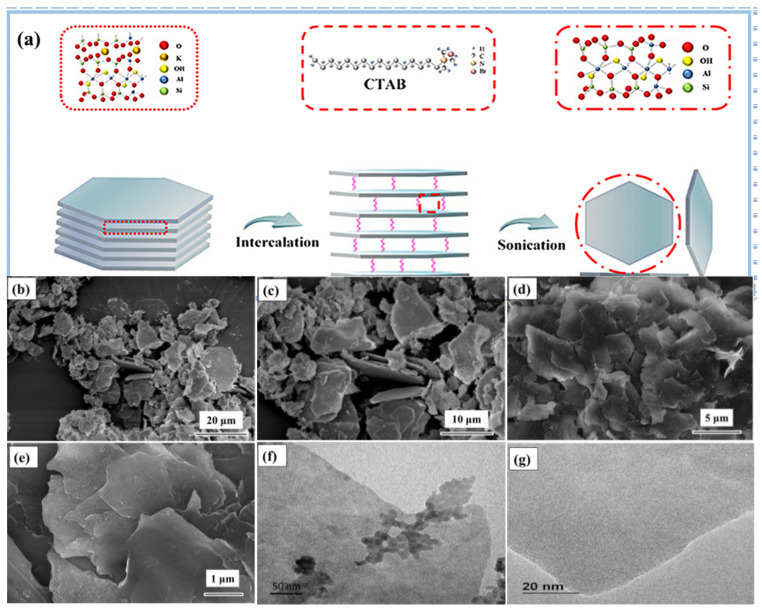
(**a**) Schematic illustration of 2D nano-mica sheet prepared by intercalation stripping method; (**b**–**e**) SEM images of nature mica powder and 2D nano-mica sheet; (**f**,**g**) TEM images of 2D nano-mica sheet.

Further, 2D nano-mica sheets were obtained by the “top-down” method from the nature mica powders [29]. The nature mica powder was firstly heated at 800 °C for an hour. Then 3 g pretreated mica powder was added into 100 mL 5 mol∙L^−1^ nitric acid under magnetic stirring at 95 °C for 5 h. The resultant product was washed by thermal deionized water until it was nearly neutral (pH value is about 7), and dried at 80 °C. Subsequently, the resultant powders were continuously reacted with NaCl supersaturated solution at 95 °C for 3 h, rinsed and filtered several times to remove excess NaCl, and dried at 80 °C. Then, we put 1.5 g of the product and 4.6 g surfactant CTAB into 150 mL deionized water and stirred at 80 °C for 24 h. The resultant product was washed with thermal deionized water to remove excess CTAB and dried at 80 °C. The intercalated mica product was further exfoliated by ultrasonication. Then, 50 mg intercalated mica was added into 50 mL absolute ethanol and ultrasonic treatment for 30 min under a sonication power of 500 W. The resultant dispersion was centrifuged at 3000 rm^−1^ for 10 min to remove the un-exfoliated CTAB-mica and get the supernatant nano-mica sheets. Finally, we centrifuged the dispersion at 10,000 rm^−1^ for 10 min and freeze-dried to obtain 2D nano-mica sheets. The 2D nano-mica sheets were prepared according to the schematic diagram shown in Figure 1a.

### 2.3. Fabrication of Nano-Mica Composite Membrane

Before use, the PVDF support membrane was first soaked with an appropriate amount of ethanol for 24 h and then soaked with an appropriate amount of deionized water for 24 h. Firstly, the collected 2D nano-mica sheets were dispersed into a certain amount of tris buffer solution (pH = 8.5, 10 mM), followed by ultrasonication for 30 min to prepare nano-mica sheets with a uniform dispersion of 0.5 mg·mL^−1^. Then, a certain amount of dopamine was dissolved into the above solution and stirred for 4 h to obtain uniform suspensions. Then, the desired amount of solution was passed through the support membrane by vacuum filtration and the attached amount of nano-mica sheets on the supporting membrane was 5 mg. The resultant composite membrane was dried at room temperature. The obtained membrane was denoted as NCM.

### 2.4. Characterization

Transmission electron microscopy (TEM, FEI TECNAI G2 F20, Hillsboro, OR, USA) was used to observe the microstructures of nano-mica sheets. The 2D nano-mica sheets were ultrasonically dispersed in ethanol and dropped on a copper mesh before the TEM test. The crystalline structure and the d-spacing of the 2D nano-mica sheets were determined using X-ray diffraction (XRD, Bruker D8 Advance, Karlsruhe, Germany) with a scan range from 1° to 80°. Dynamic light scattering (DLS, Zetasizer Nano ZS90, Malvern Panalytical, Malvern, UK) was employed to measure the particle size and zeta potential of the 2D nano-mica sheet as well as the size of the oil droplet. The oil-in-water emulsions were further analyzed by optical microscope. The surface and cross-section microscopy of the membrane was obtained by scanning electron microscopy (SEM, Hitachi S-4800, Tokyo, Japan). Before SEM measurement, the membrane samples were coated with gold for 50 s to enhance the electro-conductivity. The thickness of two-dimensional mica nanosheets was measured by atomic force microscope (AFM, JPK Nano Wizard 4, Karlsruhe, Germany) under tap mode. The water and underwater oil contact angle of the membrane were acquired from a contact angle meter (Kruss DSA-100, Hamburg, Germany) at room temperature. The membrane was placed in a special four-sided transparent vessel to measure the underwater contact angle. The oil concentration of feed and filtrate was analyzed by a total organic carbon analyzer (TOC, Shimadzu TOC-L CPN, Tokyo, Japan).

### 2.5. Preparation of Oil-in-Water Emulsions

Three kinds of oils with different viscosities, including soybean oil, mineral oil and pump oil, were selected as the oil phases. The surfactant-stabilized oil-in-water emulsions were prepared by adding oil into the water with a volume ratio of 1:99, followed by the addition of sodium dodecyl sulfate (SDS) (200 mg·L^−1^) to the mixtures. The mixtures were then stirred under 2000 rpm for 3 h to obtain homogeneous emulsions.

### 2.6. Membrane Performance

The oil/water separation performance of the NCM was tested by self-assembled laboratory equipment. The effective membrane area of the separation experiment was 15.89 cm^2^. The emulsion was filtered at about 500 mbar pressure. The permeation flux was calculated by Equation (1):

(1) $$J=\frac{V}{A\Delta tP}$$

where *J* is the permeation flux (L∙m^−2^∙h^−1^·bar^−1^), *V* is filtrate volume (L), *A* is the effective membrane area (m^2^), Δ*t* is filter time (h), *P* is the applied pressure (bar), respectively.

The oil removal rate *R* was calculated by Equation (2):

(2) $$R=\frac{C_{0}-C_{1}}{C_{0}}\times 100\%$$

where *C*_0_ and *C*_1_ respects the oil concentrations of inlet and outlet obtained by TOC, respectively.

## 3. Results

### 3.1. Preparation and Characterization of 2D Nano-Mica Sheet

Figure 1a illustrates the process of preparing 2D nano-mica sheets by intercalation stripping. In this process, a cationic surfactant can increase the interlayer spacing and weaken the interlayer force, and then it was striped into 2D nano-mica sheets by ultrasound. Before stripping, the nature mica powder has an irregular granular structure with a diameter of several microns to tens of microns, and it is almost difficult to observe the complete lamellar structure (Figure 1b,c). After stripping, the size of the mica sheet is significantly reduced (mostly in hundreds of nanometers to a few microns), and the mica sheet is in the shape of a thin sheet with obvious stratification (Figure 1d,e). The TEM images in Figure 1f,g demonstrate that the surface of the stripped mica sheet is smooth and complete, in a translucent film state, and the mica sheet layer is extremely thin [29].

**Figure 2:**
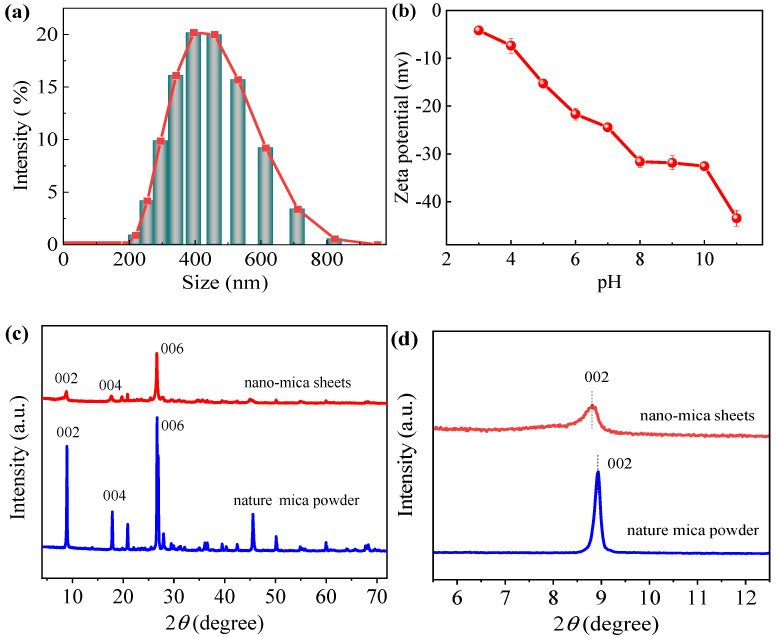
(**a**) The particle size distribution of 2D nano-mica sheet, (**b**) The zeta potential of 2D nano-mica sheet at different pH, (**c**,**d**) XRD spectra of nature mica powder and 2D nano-mica sheet.

The thickness of the 2D nano-mica sheet was measured by an atomic force microscope, and its thickness was about 1.0 nm (Figure S1). The results of DLS from Figure 2a showed that the size of the 2D nano-mica sheet is mainly 200–800 nm, which is consistent with the results of SEM and TEM. The zeta potential test of the 2D nano-mica sheet (Figure 2b) indicates that the nano-mica sheet is significantly negative at different pH values, and its zeta potential gradually decreases with the increase of pH value. In order to visually observe the dispersion stability of the exfoliated 2D nano-mica sheets, the mica nanosheets were ultrasonically dispersed in ethanol to form a homogeneous dispersion of 20 mg·L^−1^, and then allowed to stand at room temperature for five months. During this period, the dispersion stability of the solution was observed by taking digital photos, and the results are shown in Figure S2. Remarkably, the 2D nano-mica sheets in ethanol produce a pronounced Tyndall phenomenon even after five months. It shows that the 2D nano-mica sheets have excellent dispersion and stability in ethanol, which is due to the rich negatively charged groups on the surface of the nanosheets.

Figure 2c XRD of nature mica powder illustrates that it mainly includes characteristic diffraction peaks of 8.94° (002), 17.86° (004), and 26.68° (006), and the peak types of diffraction peaks are relatively sharp, indicating that the nature mica powder has high crystallinity. After intercalation stripping, the diffraction peak position of the 2D nano-mica sheet is basically consistent with that of nature mica powder, but the diffraction peak intensity decreases significantly, indicating that the crystal structure of mica is not damaged, but there is decrease of thickness after stripping [31]. Figure 2d shows that the characteristic diffraction peak of the two-dimensional nano-mica sheet (002) after stripping is reduced from 8.94° of the nature mica powder to 8.82°. Meanwhile, the half-peak width of the characteristic peak of the 002 crystal plane of the 2D nano-mica sheet becomes wider after stripping, which indicates that the mica sheet is successfully stripped, and its lamella becomes thinner and the lamella spacing becomes larger after stripping [32].

### 3.2. Preparation and Characterization of Membranes

**Figure 3:**
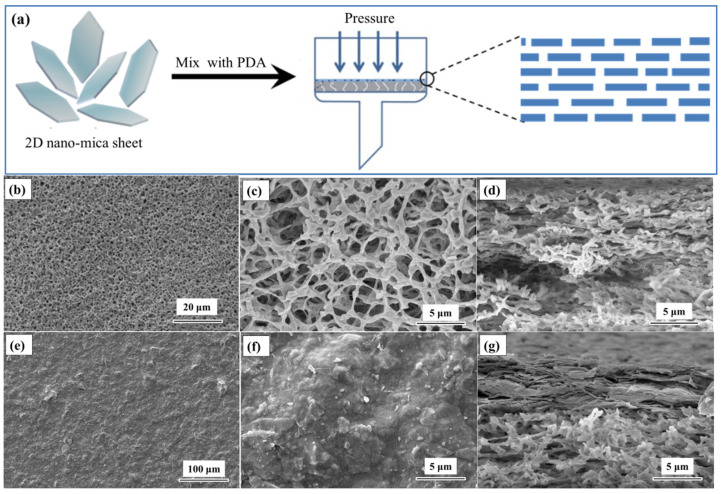
(**a**) Schematic diagram of the assembly process of NCM; (**b**,**c**) SEM images of PVDF membrane surface, (**d**) SEM images of PVDF membrane cross sectional, (**e**,**f**) SEM images of NCM surface, (**g**) SEM images of NCM cross sectional.

The NCM was fabricated by filtering the 2D nano-mica sheets and dopamine suspensions on the PVDF membrane (Figure 3a). Then, 2D nano-mica sheets were co-deposited with dopamine on PVDF substrate to prepare NCM by vacuum-assisted filtration. The self-polymerization of dopamine is a simple reaction of enzymatic oxidation. Dopamine may be oxidized to dopamine-quinone, followed by intermolecular interaction, which may be the main reason for its easy attachment to inorganic and organic substrates [33]. The PVDF membranes were modified by dopamine under mild polymerization conditions, which not only improved the hydrophilicity of the membranes, but also acted as a cross-linking agent, allowing the 2D nano-mica sheets to adhere more firmly to the PVDF substrate [34,35]. The chemical compositions of the 2D nano-mica sheet, PVDF membrane, PDA coated PVDF and NCM were characterized by FTIR, as shown in Figure S3. FTIR spectra confirmed the successful coating of PDA and 2D nano-mica sheet on the PVDF membrane surface. To intuitively reflect the distribution of mica sheets in PVDF and the morphological changes of composites before and after composite, the surface micromorphology of PVDF membrane and NCM was observed by SEM, as shown in Figure 3b–g. Figure 3b,c show that the PVDF membrane presents a typical fibrous structure, and the membrane surface is relatively flat and rich in pores. The PVDF membrane also has an abundant three-dimensional network pore structure on the side (Figure 3d). The surface of the NCM is extremely rough, with many irregular layered protrusions. It is observed that many-layered nano-mica sheets are attached to the side of the PVDF membrane (Figure 3e–g), which proves that the nano-mica sheets are successfully combined with the PVDF membrane. In addition, the surface roughness of the membrane was analyzed by AFM, and the surface roughness of the NCM was 24.3 nm (Figure S4).

The wettability of the membrane has a significant impact on the separation performance [36]. The wettability of the membrane was evaluated by static and dynamic contact angle tests. Figure S5 shows that the water contact angle of the PVDF base membrane in the air is 128°, and the oil contact angle underwater is 128.9°. The water contact angle of NCM loaded with nano-mica sheet in the air is 20.8°, and the oil contact angle underwater is 151.4°, indicating that NCM has excellent hydrophilicity and underwater super oil repellency. When oily wastewater reaches above the membrane interface, water can quickly wet the membrane and continue to penetrate downward, while oil droplets are blocked above the membrane interface, and finally, achieve the purpose of separating oily wastewater. Figure S6 is the dynamic oil contact angle underwater environment of NCM. When oil droplets were rolled close to the membrane surface, oil droplets could not be adhered by the NCM, which shows that it has excellent oil adhesion resistance in a water environment. To further prove the oil adhesion resistance of the NCM underwater, the NCM was obliquely immersed in water, and then dichloromethane (oil red staining) was dropped onto the membrane surface. Dichloromethane stained with oil red will roll down from the NCM rapidly (Video 1). Similarly, the PVDF membrane was obliquely immersed in water, and then dichloromethane (oil red staining) was dropped onto the membrane surface, and dichloromethane will adhere to the PVDF membrane (Video 2). Due to the hydrophilicity and underwater oil repellency of the NCM, it has the potential to separate oil and water by intercepting oil droplets [37].

**Figure 4:**
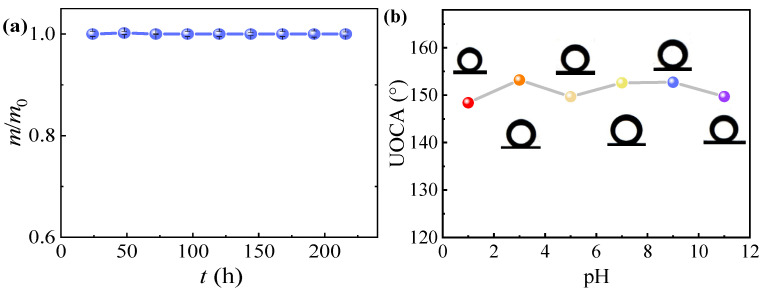
(**a**) Weight loss of NCM after immersion in water for different time, (**b**) underwater oil contact angel (UOCA) at different pH value.

In the process of emulsion separation, the stability of the membrane is also one of the important factors affecting its practical application. In order to study the physical stability of the NCM, the membrane was completely immersed in water for 216 h, the weight of the membrane was measured every 24 h, and the material loss on the membrane was evaluated nine times. As shown in Figure 4a, there is almost no weight loss after the membrane is soaked for 216 h, indicating that the NCM has excellent stability in water. The prepared NCM was immersed in the solution with a pH value of 1–11 for 24 h, and the changes of underwater oil contact angle were measured. Figure 4b shows that pH value has no significant effect on the underwater oil contact angle of the membrane. The results show that nano-mica sheets can stably adhere to the membrane under different pH values and maintain good oil repellency, which provides a basis for the practical application of membrane oil-water separation.

### 3.3. The Separation Performance of Oil-in-Water Emulsion

**Figure 5:**
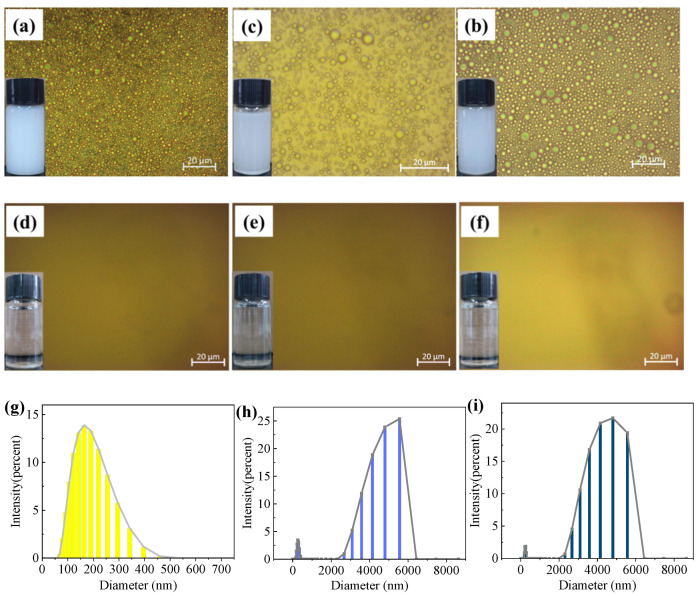
Optical microscope and droplet size distribution images of SDS-stabilized oil-in-water emulsions: (**a**,**d**,**g**) soya-bean oil, (**b**,**e**,**h**) mineral oil and (**c**,**f**,**i**) pump oil.

**Figure 6:**
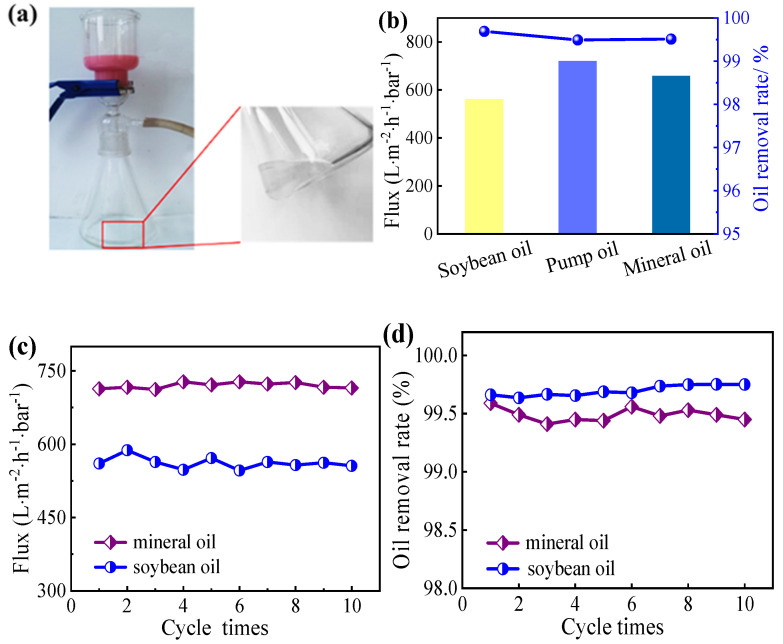
(**a**) Oil/water separation experimental device of the NCM, (**b**) flux and oil removal rate of the SDS-stabilized oil-in-water emulsions (soya-bean oil, mineral oil and pump oil), (**c**) flux changes of emulsified soybean oil and pump oil for 10 filtration cycles, (**d**) oil removal rate changes of emulsified soybean oil and pump oil for 10 filtration cycles.

The optical microscope and droplet size distribution images of SDS-stabilized oil-in-water emulsions and filtration solutions: soya-bean oil (a, d, g), mineral oil (b, e, h) and pump oil (c, f, i) are exhibited in Figure 5. The three initial SDS-stabilized oil-in-water emulsions were all milky white with numerous micron and submicron oil droplets. No droplets could be observed in the corresponding optical microscopic images after the separation, which further confirmed the high separation efficiency of the membrane. The three solutions after filtration were completely clarified, and no droplets were observed in the corresponding optical microscopy images, which confirmed the high separation efficiency of the NCM. The oil-water separation experiment of the NCM was carried out on a vacuum suction filtration device (Figure 6a). In addition, the size distribution of droplets in the emulsion was measured by DLS. The three emulsion droplets have wide oil droplet size distribution, and their diameters range from hundreds of nanometers to thousands of nanometers.

The flux and separation efficiency of NCM for SDS-stabilized oil-in-water emulsions (soya-bean oil, mineral oil and pump oil) were investigated, and the results were displayed in Figure 6b. The flux range of NCM for SDS-stabilized oil-in-water emulsions (soya-bean oil, mineral oil and pump oil) is 560–720 L∙m^−2^∙h^−1^·bar^−1^. The flux difference between the three emulsions is attributed to the different viscosity of oils [38,39]. The oil removal rate of NCM for all surfactant stabilized oil-in-water emulsions is close to 100% (99.7% for soybean oil, 99.5% for pump oil, and 99.5% for mineral oil), and the NCM has outstanding oil-water separation performance for SDS-stabilized oil-in-water emulsions. The outstanding oil-water separation performance of NCM can be attributed to the low water transport resistance of the densely packed hydrophilic 2D nano-mica sheet layer on membrane. As a comparison, the PVDF membrane was used to remove emulsified soybean oil emulsion in water. Since PVDF membrane is hydrophobic, it cannot be used for oil-water emulsion separation.

The reusability or long-term operation performance of NCM is critical for cost control in practical applications. To further evaluate the structural stability and anti-pollution performance of the NCM oil-water separation membrane during long-term operation, SDS-emulsified mineral oil and soya-bean oil were selected for 10 filtration cycle stability tests, and the results are shown in Figure 6c,d. After 10 filtration cycles of continuous test, the flux and oil removal ratios of the NCM did not decrease significantly, which demonstrates that the NCM has excellent oil-water separation stability performance. Moreover, the high separation performance of the NCM towards oil/water emulsion at different pH environments (Figure S7) further confirmed its good stability.

Table 1 compares the separation performance of the NCM with other membranes for similar applications. The nano-mica composite membranes exhibit higher competitiveness, which could potentially be applied for the filtration with surfactant-stabilized oil-in-water emulsion.

## 4. Conclusions

In summary, the NCM with effective oil-water separation performance was successfully constructed by using the 2D nano-mica sheet through a simple blending suction filtration method. The NCM is hydrophilic in air and super-oleophobic underwater (the water contact angle in the air is 37.6°, and the oil contact angle in water is 151.4°). The NCM has excellent stability in the water environment and very low adhesion to oil. The pH value also had no significant effect on the underwater oil contact angle of the NCM. In addition, the oil removal performance of the NCM for the SDS-stabilized oil (soya-bean oil, mineral oil and pump oil) -in-water emulsions reached more than 99.5%, and the oil removal performance did not decrease significantly after 10 filtration cycles. This study provides a new idea for the development of efficient oil-water separation materials and reveals the potential application value of mica in the field of energy and the environment.

## Figures and Tables

**Table 1:** Comparison of the separation performance among various membranes.

Membrane	Flux (L·m^−2^·h^−1^·bar^−1^)	Oil Removal Rate (%)	Reference
PVDF@pDA@SiO_2_ nanocomposite membrane	572	98	[34]
PA6(3)T membrane	321	92.7	[40]
PEM-coated membrane	24	99.98	[41]
PEM based NF membranes	/	>99%	[42]
RGO membrane	/	99.6%	[43]
Al_2_O_3_/AC hybrid membrane	~1.85	99.44	[44]
Al_2_O_3_ membrane	~1.55	98.2	[44]
NCM	~720	99.5	This work

## Data Availability

Not applicable.

## Supplementary Materials

The following supporting information can be downloaded at: https://www.mdpi.com/article/10.3390/nano12172895/s1, Figure S1: AFM images of 2D nano-mica sheet; Figure S2: Photographs of 2D nano-mica sheet s dispersions in ethanol; Figure S3: FTIR spectra of the 2D nano-mica sheet, PVDF membrane, PDA coated PVDF and NCM; Figure S4: AFM images of the NCM; Figure S5: The oil contact angle underwater and water contact angle in the air of membrane: (a) PVDF membrane, (b) nano-mica composite membranes; Figure S6: Dynamic oil contact angle underwater environment of nano-mica composite membranes; Figure S7: Mineral oil/water separation properties of the NCM at different pH values; Video 1: Super-oleophobic in water of NCM; Video 2: Lipophilic in water of PVDF membrane; Video 3: Separation of oil-water emulsion by PVDF membrane.

## References

[1] Miller D.J., Dreyer D.R., Bielawski C.W., Paul D.R., Freeman D.B. (2017). Oberflächenmodifizierung von Wasseraufbereitungsmembranen. Angew. Chem..

[2] Adebajo M., Frost R.L., Kloprogge T., Carmody O., Kokot S. (2003). Porous materials for oil spill cleanup: A review of synthesis and absorbing properties. J. Porous Mater..

[3] Zhang X., Li Z., Liu K., Jiang L. (2013). Bioinspired Multifunctional Foam with Self-Cleaning and Oil/Water Separation. Adv. Funct. Mater..

[4] Zhang S., Wang P., Fu X., Chung T.-S. (2014). Sustainable water recovery from oily wastewater via forward osmosis-membrane distillation (FO-MD). Water Res..

[5] Peterson C.H., Rice S.D., Short J.W., Esler D., Bodkin J.L., Ballachey B.E., Irons D.B. (2003). Long-term ecosystem response to the Exxon Valdez oil spill. Science.

[6] Cheryan M., Rajagopalan N. (1998). Membrane processing of oily streams. Wastewater treatment and waste reduction. J. Membr. Sci..

[7] Liu Y.-N., Su Y., Guan J., Cao J., Zhang R., He M., Jiang Z. (2018). Asymmetric Aerogel Membranes with Ultrafast Water Permeation for the Separation of Oil-in-Water Emulsion. Acs Appl. Mater. Interfaces.

[8] Shannon M.A., Bohn P.W., Elimelech M., Georgiadis J.G., Mariñas B.J., Mayes A.M. (2008). Science and technology for water purification in the coming decades. Nature.

[9] Xue Z., Wang S., Lin L., Chen L., Liu M., Feng L., Jiang L. (2011). A Novel Superhydrophilic and Underwater Superoleophobic Hydrogel-Coated Mesh for Oil/Water Separation. Adv. Mater..

[10] Lee C., Baik S. (2010). Vertically-aligned carbon nano-tube membrane filters with superhydrophobicity and superoleophilicity. Carbon.

[11] Thangavelu K., Ravaux F., Zou L.D. (2021). Cellulose acetate-MoS_2_ amphiphilic Janus-like fibrous sponge for removing oil from wastewater. Environ. Technol. Innov..

[12] Liu S., Wang S.S., Wang H., Lv C.J., Miao Y.C., Chen L., Yang S.D. (2020). Gold nanoparticles modified graphene foam with superhydrophobicity and superoleophilicity for oil-water separation. Sci. Total Environ..

[13] Thangavelu K., Aubry C., Zou L.D. (2021). Amphiphilic Janus 3D MoS_2_/rGO Nanocomposite for Removing Oil from Wastewater. Ind. Eng. Chem. Res..

[14] Li N., Yue Q., Gao B., Xu X., Su R., Yu B. (2018). One-step synthesis of peanut hull/graphene aerogel for highly efficient oil-water separation. J. Clean. Prod..

[15] Belfort G. (2019). Membrane Filtration with Liquids: A Global Approach with Prior Successes, New Developments and Unresolved Challenges. Angew. Chem..

[16] Padaki M., Murali R.S., Abdullah M.S., Misdan N., Moslehyani A., Kassim M.A., Hilal N., Ismail A.F. (2015). Membrane technology enhancement in oil-water separation. A review. Desalination.

[17] Pendergast M.M., Hoek E.M. (2011). A review of water treatment membrane nanotechnologies. Energy Environ. Sci..

[18] YNie Y., Zhang S., He Y., Zhang L., Wang Y., Li S., Wang N. (2022). One-step modification of electrospun PVDF nanofiber membranes for effective separation of oil–water emulsion. New J. Chem..

[19] ZXue Z., Cao Y., Liu N., Feng L., Jiang L. (2014). Special wettable materials for oil/water separation. J. Mater. Chem. A.

[20] Wang B., Liang W., Guo Z., Liu W. (2015). Biomimetic super-lyophobic and super-lyophilic materials applied for oil/water separation: A new strategy beyond nature. Chem. Soc. Rev..

[21] Pan T.D., Li Z.J., Shou D.H., Shou W., Fan J.T., Liu X., Liu Y. (2019). Buoyancy Assisted Janus Membrane Preparation by ZnO Interfacial Deposition for Water Pollution Treatment and Self-cleaning. Adv. Mater. Interfaces.

[22] Fen L., Zhang Z., Mai Z., Ma Y., Liu B., Jiang L., Zhu D. (2004). A super-hydrophobic and super-oleophilic coating mesh film for the separation of oil and water. Angew. Chem..

[23] Zhu Z., Wang W., Qi D., Luo Y., Liu Y., Xu Y., Cui F., Wang C., Chen X. (2018). Calcinable Polymer Membrane with Revivability for Efficient Oily-Water Remediation. Adv. Mater..

[24] Ou X., Yang X., Zheng J., Liu M. (2019). Free-Standing Graphene Oxide-Chitin Nanocrystal Composite Membrane for Dye Adsorption and Oil/Water Separation. Acs Sustain. Chem. Eng..

[25] Yang J., Zhang Z., Xu X., Zhu X., Men X., Zhou X. (2012). Superhydrophilic-superoleophobic coatings. J. Mater. Chem..

[26] Tareen A.K., Khan K., Aslam M., Liu X., Zhang H. (2021). Confinement in two-dimensional materials: Major advances and challenges in the emerging renewable energy conversion and other applications. Prog. Solid State Chem..

[27] Sui X., Yuan Z., Yu Y., Goh K., Chen Y. (2020). 2D Material Based Advanced Membranes for Separations in Organic Solvents. Small.

[28] Ahmed Z., Rehman F., Ali U., Ali A., Iqbal M., Thebo K.H. (2021). Recent Advances in MXene-based Separation Membranes. Chembioeng Rev..

[29] Pan X.-F., Gao H.-L., Lu Y., Wu C.-Y., Wu Y.-D., Wang X.-Y., Pan Z.-Q., Dong L., Song Y.-H., Cong H.-P. (2018). Transforming ground mica into high-performance biomimetic polymeric mica film. Nat. Commun..

[30] Ying W., Han B., Lin H., Chen D., Peng X. (2019). Laminated mica nanosheets supported ionic liquid membrane for CO_2_ separation. Nanotechnology.

[31] Ding J., Zhao H., Yu H. (2020). Superior to graphene: Super-anticorrosive natural mica nanosheets. Nanoscale.

[32] Jiang H., Jiang L., Zhang P., Zhang X., Ma N., Wei H. (2021). Force-Induced Self-Assembly of Supramolecular Modified Mica Nanosheets for Ductile and Heat-Resistant Mica Papers. Langmuir.

[33] Liu Y., Ai K., Lu L. (2014). Polydopamine and its derivative materials: Synthesis and promising applications in energy, environmental, and biomedical fields. Chem. Rev..

[34] Cui J., Zhou Z., Xie A., Meng M., Cui Y., Liu S., Lu J., Zhou S., Yan Y., Dong H. (2019). Bio-inspired fabrication of superhydrophilic nanocomposite membrane based on surface modification of SiO_2_ anchored by polydopamine towardseffective oil-water emulsions separation. Sep. Purif. Technol..

[35] Zhang P.-B., Liu C.-J., Sun J., Zhu B.-K., Zhu L.-P. (2017). Fabrication of composite nanofiltration membranes by dopamine-assisted poly (ethylene imine) deposition and cross-linking. J. Zhejiang Univ.-Sci. A.

[36] Yang X., Sun H., Pal A., Bai Y., Shao L. (2018). Biomimetic Silicification on Membrane Surface for Highly Efficient Treatments of Both Oil-in-Water Emulsion and Protein Wastewater. ACS Appl. Mater. Interfaces.

[37] Tao M., Xue L., Liu F., Jiang L. (2014). An Intelligent Superwetting PVDF Membrane Showing Switchable Transport Performance for Oil/Water Separation. Adv. Mater..

[38] Gao S.J., Shi Z., Bin Zhang W., Zhang F., Jin J. (2014). Photoinduced Superwetting Single-Walled Carbon Nanotube/TiO_2_ Ultrathin Network Films for Ultrafast Separation of Oil-in-Water Emulsions. ACS Nano.

[39] Yang W., Pan M., Zhang J., Zhang L., Lin F., Liu X., Huang C., Chen X., Wang J., Yan B. (2021). A Universal Strategy for Constructing Robust and Antifouling Cellulose Nanocrystal Coating. Adv. Funct. Mater..

[40] Lin Y.-M., Rutledge G.C. (2018). Separation of oil-in-water emulsions stabilized by different types of surfactants using electrospun fiber membranes. J. Membr. Sci..

[41] Zhang G., Li L., Huang Y., Hozumi A., Sonoda T., Su Z. (2018). Fouling-resistant membranes for separation of oil-in-water emulsions. RSC Adv..

[42] Virga E., Parra M.A., de Vos W.M. (2021). Fouling of polyelectrolyte multilayer based nanofiltration membranes during produced water treatment: The role of surfactant size and chemistry. J. Colloid Interface Sci..

[43] Liu N., Zhang M., Zhang W., Cao Y., Chen Y., Lin X., Xu L., Li C., Feng L., Wei Y. (2015). Ultralight free-standing reduced graphene oxide membranes for oil-in-water emulsion separation. J. Mater. Chem. A.

[44] Fard A.K., Bukenhoudt A., Jacobs M., McKay G., Atieh M.A. (2018). Novel hybrid ceramic/carbon membrane for oil removal. J. Membr. Sci..

