# Supplementary material for: 2D Nano-Mica Sheets Assembled Membranes for High-Efficiency Oil/Water Separation
Source: Nanomaterials (Basel). 2022 Aug 23;12(17):2895. doi: 10.3390/nano12172895 (PMC9457926; doi:10.3390/nano12172895)
Supplement: Supplementary file 1 [file nanomaterials-12-02895-s001.zip › Supporting Information.pdf]

2D nano-mica sheets assembled membranes for high-efficient oil/water separation

Yan Bao [1,3,†], Bin Wang [2,†], Conghui Du [1], Qiuhui Shi [1], Wenlong Xu [3] and Zhining Wang [1,*]

[1] Shandong Key Laboratory of Environmental Processes and Health, School of Environmental Science and Engineering, Shandong University, Qingdao 266237, China
[2] Informatization Office, Shandong University, Ji'nan 250100, China
[3] Qingdao Institute of Bioenergy and Bioprocess Technology, Chinese Academy of Sciences, Qingdao 266101, China
[*] Correspondence: wangzhn@sdu.edu.cn
[†] These authors contributed equally.

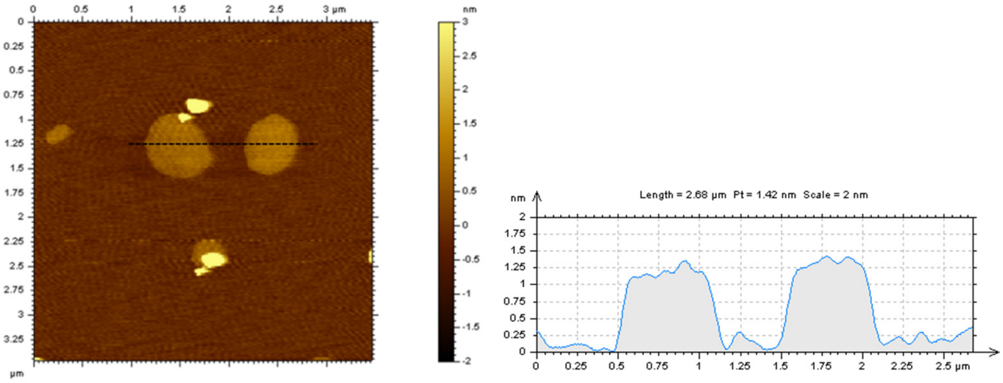

Figure S1. AFM images of 2D nano-mica sheet

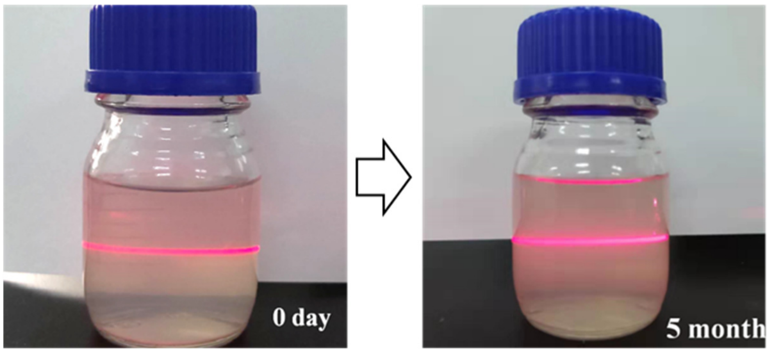

Figure S2. Photographs of 2D nano-mica sheet s dispersions in ethanol

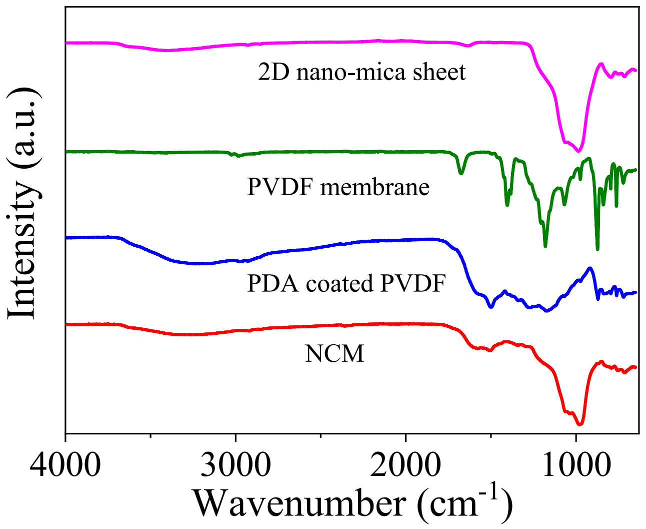

Figure S3. FTIR spectra of the 2D nano-mica sheet, PVDF membrane, PDA coated PVDF and NCM

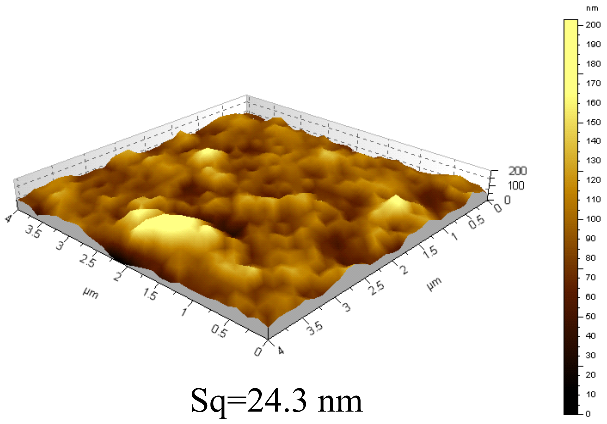

Figure S4. AFM images of the NCM

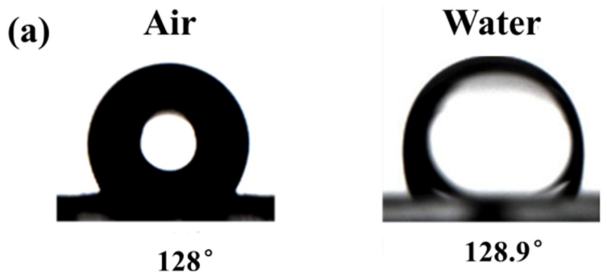

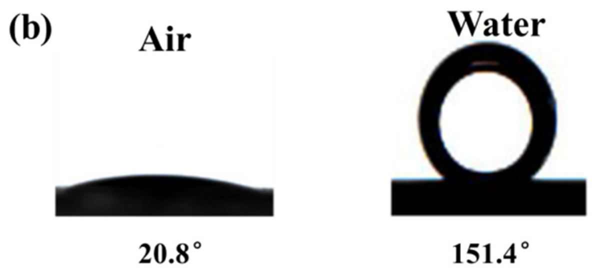

Figure S5. The oil contact angle underwater and water contact angle in the air of membrane: (a) PVDF membrane, (b) nano-mica composite membranes

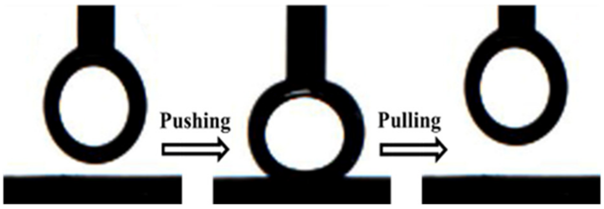

Figure S6. Dynamic oil contact angle underwater environment of nano-mica composite membranes

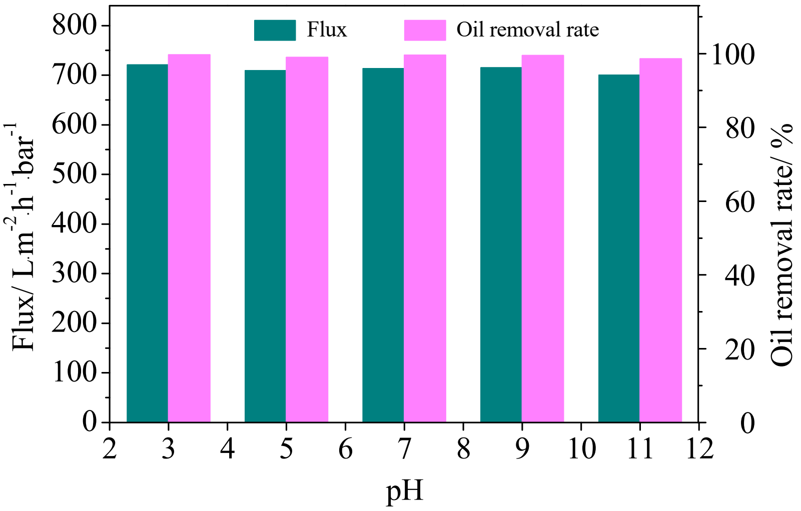

Figure S7. Mineral oil/water separation properties of the NCM at different pH values
